# Rest-related consolidation protects the fine detail of new memories

**DOI:** 10.1038/s41598-018-25313-y

**Published:** 2018-05-01

**Authors:** Michael Craig, Michaela Dewar

**Affiliations:** Department of Psychology, School of Social Sciences, Heriot-Watt University, Edinburgh, EH14 4AS United Kingdom

## Abstract

Newly encoded memories are labile and consolidate over time. The importance of sleep in memory consolidation has been well known for almost a decade. However, recent research has shown that awake quiescence, too, can support consolidation: people remember more new memories if they quietly rest after encoding than if they engage in a task. It is not yet known how exactly this rest-related consolidation benefits new memories, and whether it affects the fine detail of new memories. Using a sensitive picture recognition task, we show that awake quiescence aids the fine detail of new memories. Young adults were significantly better at discriminating recently encoded target pictures from similar lure pictures when the initial encoding of target pictures had been followed immediately by 10 minutes of awake quiescence than an unrelated perceptual task. This novel finding indicates that, in addition to influencing how much we remember, our behavioural state during wakeful consolidation determines, at least in part, the level of fine detail of our new memories. Thus, our results suggest that rest-related consolidation protects the fine detail of new memories, allowing us to retain detailed memories.

## Introduction

In order for labile new memories to be remembered over the longer term they must be consolidated, i.e. strengthened and stabilised, over time^1,2^. Research in humans and non-human animals suggests that an important mechanism of consolidation is the automatic neural reactivation of newly encoded memories in the minutes that follow their formation^3–15^. Memory reactivation is observed especially during periods of post-encoding sleep and awake quiescence (quiet resting), where the magnitude of reactivation predicts subsequent memory^3,4,6,9,16^, and the electrophysiological disruption of reactivation results in reduced memory^11^. Sleep and awake quiescence may be especially conducive to memory reactivation due to reductions in sensory input and task engagement, which could otherwise impact this process^16–18^.

Behavioural research in humans supports this hypothesis, demonstrating better memory retention after post-encoding sleep^19–22^ and post-encoding awake quiescence^18,23–25^, relative to a filled period of wakefulness. While the effect of sleep in memory is well established, research into the effects of awake quiescence is still in its infancy. To date the latter research has shown that humans retain more words and associative information after a 15–30 minute delay^23–25^ and a 7-day delay^26,27^ when they rest quietly in the minutes immediately after memory encoding than when they engage in an unrelated perceptual task. This effect cannot be explained away by intentional rehearsal during the rest period, because people remember more memories after awake quiescence than task conditions, even when the memories cannot be rehearsed intentionally^26,27^. Thus, converging human and animal research indicates that awake quiescence supports the offline consolidation of new memories.

It is not yet known how exactly this rest-related consolidation benefits new memories, and whether it affects the fine detail of new memories. Rest-related consolidation could increase the number of retained memories without necessarily supporting the fine detail of individual memories. Therefore, in the study reported here, we examined how a brief period of awake quiescence after encoding affects the fine detail of new memories. To this end, we applied the robust and sensitive ‘Mnemonic Similarity Task’ (MST)^28–30^ to probe the fine detail of new memories. The MST, originally designed to assess pattern separation, is sensitive to manipulations during memory encoding and consolidation^30,31^. In this task, participants are first sequentially presented a set of photos of everyday items (targets). Memory for the presented items is subsequently examined via a visual recognition test, where participants are sequentially presented a set of photos of everyday items again, but asked to respond “old”, “similar”, or “new” to indicate whether each presented item is (i) identical to a photo presented during encoding (target, correct response = “old”), (ii) visually similar, but subtly different, to a photo presented during encoding (lure, correct response = “similar”), or (iii) a new photo that was not presented during encoding (foil, correct response = “new”). An equal number of target, lure and foil photos are presented. Two memory measures are typically extracted from the MST: a standard recognition score, and a Lure Discrimination Index (LDI) score. The standard recognition score is calculated as the proportion of “old” responses to target items minus the proportion of “old” responses to foils, and thus reflects a person’s ability to correctly endorse targets, while rejecting foils. A high standard recognition score can indicate good gist-based memory, but it need not imply that memories are fine in detail^32,33^. The LDI score is calculated as the proportion of “similar” responses to lures minus the proportion of “similar” responses to foils, and thus reflects a person’s ability to discriminate lures from targets, while controlling for any response biases^28,34^. Thus, if a participant holds detailed representations of encoded target photos, they should demonstrate superior ability to correctly identify subtle visual differences in subsequently presented lures, and thus discriminate them from targets (i.e. respond “similar” to lures, rather than “old”). However, if a participant holds less detailed representations of encoded target photos, they should be less likely to correctly identify subtle visual differences in subsequently presented lures, and thus incorrectly mistake lures for target items (i.e. respond “old” to lures, rather than “similar”). Thus, a higher LDI score should indicate memories of fine detail, where subtle visual differences between previously encoded targets and presented lures can be identified.

Using the MST, Borota *et al*.^30^ recently demonstrated a positive dose-dependent effect of post-encoding caffeine administration on LDI scores. They hypothesised that caffeine promotes the consolidation of encoded (target) items, thus supporting participants’ ability to discriminate these viewed (target) items from similar (lure) items during subsequent testing^30^. Similarly, we anticipated that if *rest-related consolidation* protects the fine detail of newly formed memories, then the MST LDI score should reveal this, because people who experience post-encoding awake quiescence should be better able to discriminate targets from lure items.

**Figure 1:**
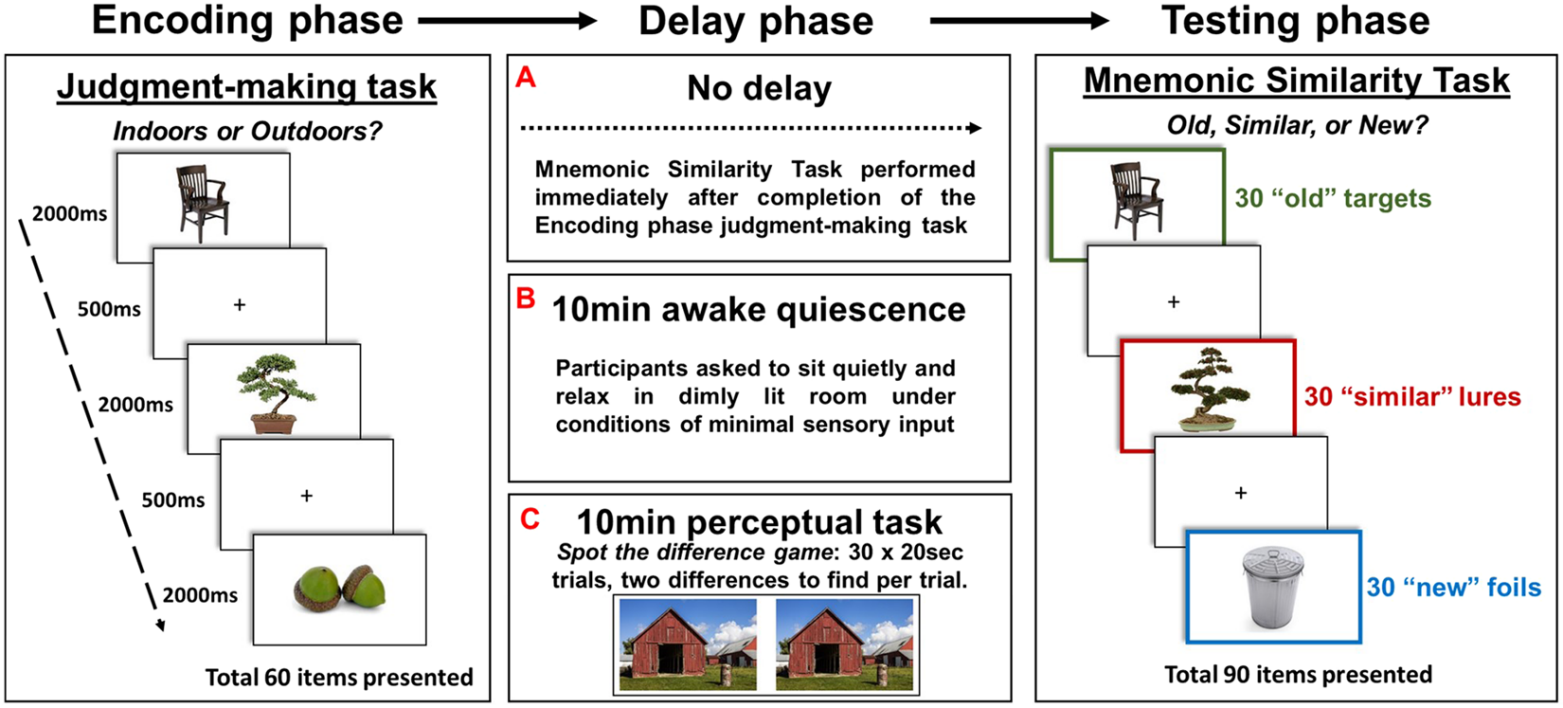
Experimental paradigm. Participants underwent three phases: (i) encoding, (ii) delay, and (iii) testing. During encoding, participants were presented 60 photos of a range of unique everyday items from the Mnemonic Similarity Task database (e.g. Stark *et al*. 2013). Participants incidentally encoded these items via the performance of a judgment making task, where they were required to respond whether a presented item would typically be found indoors or outdoors. Each item was presented for 2000 ms and was followed by a 500 ms inter-stimulus crosshair (+). Following encoding, participants completed one of three delay conditions: (**A**) no delay (N = 20), (**B**) 10 minutes of awake quiescence (N = 20), or (**C**) 10 minutes of an engaging perceptual task (spot-the-difference game) (N = 20). In the subsequent testing phase, participants were presented 30 of the ‘old’ items presented during encoding (targets), along with 30 ‘similar’ items that were visually similar objects from the same semantic category to the remaining 30 items presented during encoding (lures), and 30 ‘new’ items that were visually and semantically different to the items presented during encoding (foils). There was no limit on the time to respond during testing.

In our between-subjects study (see Fig. 1), sixty young adults were exposed to a subset of 60 photos of everyday items from the MST within the context of an incidental encoding task (indoor/outdoor judgement making task), before experiencing either: (i) no delay between item encoding and the subsequent testing phase *(‘No delay group’*, N = 20), (ii) 10 minutes of awake quiescence, i.e. resting quietly in dimly-lit room *(‘Awake quiescence delay group’*, N = 20), or (iii) 10 minutes of a perceptual task, i.e. playing a spot-the-difference game (*‘Perceptual task delay group’*, N = 20). Participants’ memory for the earlier presented items was then probed via the MST memory test (see above), during which participants were presented 90 photos of everyday items: 30 were *old*, i.e. were identical to those presented during encoding (targets), 30 were *similar* (visually similar items from the same semantic category) to the remaining photos presented during encoding (lures), and 30 were *new* to those presented during encoding (foils) - see Fig. 1 for examples. Using the responses in the MST memory test we calculated a standard recognition score - to measure gist memory - and an LDI score - to measure the fine detail of memories. We also measured response times during the MST memory test and during the encoding task. We hypothesised that, if post-encoding awake quiescence protects the fine detail of new memories, participants’ LDI scores should be superior following the awake quiescence delay than the perceptual task delay.

## Results

**Figure 2:**
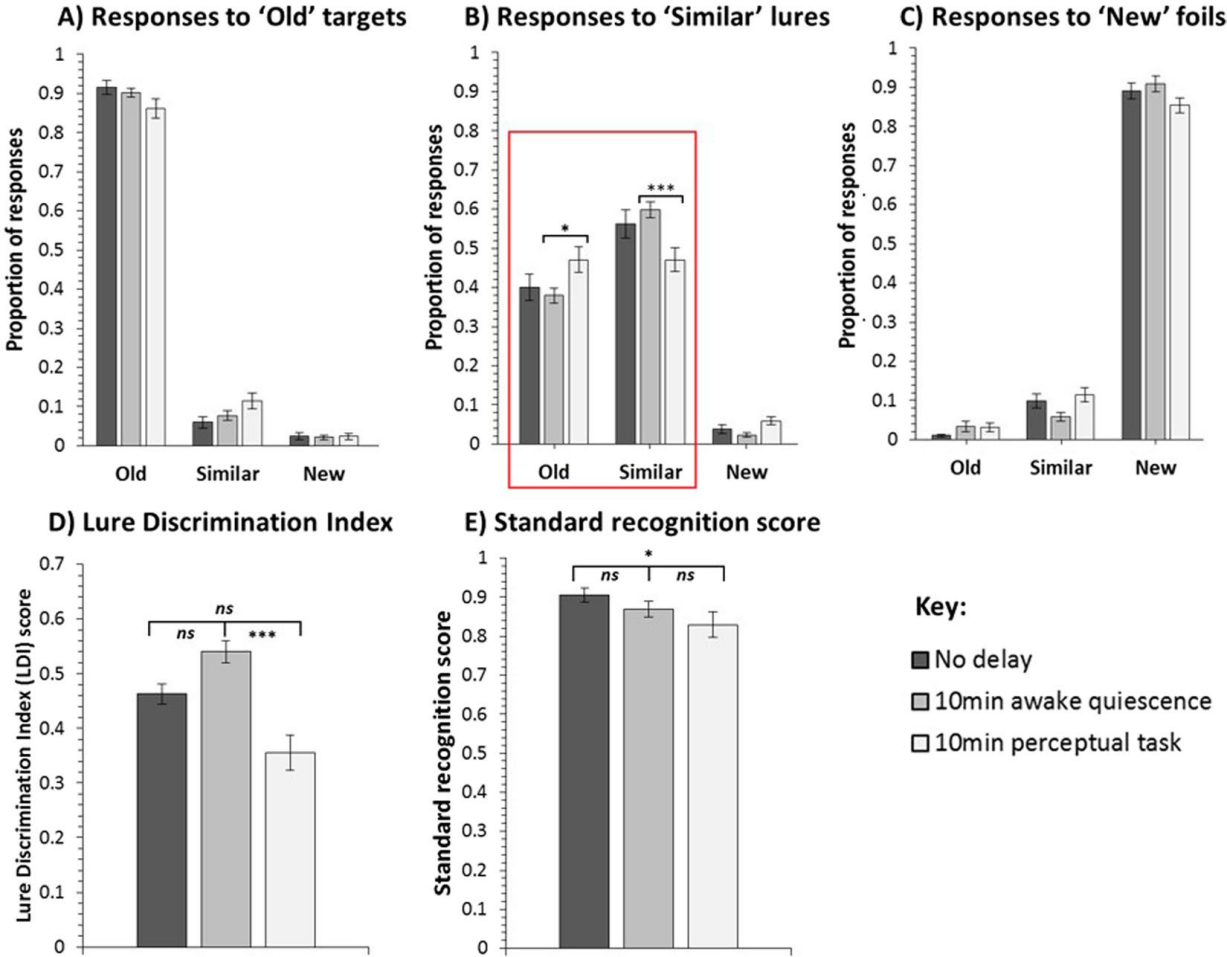
Mnemonic Similarity Test (MST) response data for the no delay (N = 19), 10 min awake quiescence delay (N = 19), and 10 min perceptual task delay (N = 18) groups showing the proportion of old, similar, and new, responses to (**A**) ‘old’ targets, (**B**) ‘similar’ lures, and (**C**) ‘new’ foils. Lure Discrimination Index (LDI) scores for the three groups are also shown (**D**) – this measure is calculated as the proportion of “similar” responses to lures minus the proportion of “similar” responses to foils, and thus reflects a person’s ability to discriminate similar lures from old targets, while controlling for any response biases. Standard recognition measure (**E**) - this measure is calculated as the proportion of “old” responses to target items minus the proportion of “old” responses to foils, and thus reflects a person’s ability to correctly endorse old targets, while rejecting new foils. The awake quiescence delay group’s ability to discriminate between targets and lures was significantly better than that of the perceptual task delay group, as shown in the groups’ (**D**) LDI scores and (**B**) specific responses to lures. The latter (see red box) shows that the perceptual task delay group provided a significantly higher proportion of (incorrect) old responses to lure items than those in the awake quiescence delay group. In contrast, the awake quiescence delay group provided a significantly higher proportion of (correct) similar responses to lure items than those in the perceptual task delay. In all cases, error bars show the standard error of the mean. Non-corrected significance levels: *p < 0.05, **p < 0.01, ***p < 0.001.

Our three delay condition groups were well matched in their demographic information (see supplementary information). Participants’ responses in the encoding task (indoor/outdoor judgment-making task) were also matched across groups (see supplementary information). Importantly, significant group differences emerged in the subsequent memory test (Mnemonic Similarity Task, MST). Participants in the delay groups differed significantly in their LDI scores, which was calculated as the proportion of “similar” responses to lures minus the proportion of “similar” responses to foils^28^, (F(2,53) = 6.954, P = 0.002, ηρ² = 0.208) - see Fig. 2D. Specifically, the LDI score was significantly higher in the awake quiescence delay than the perceptual task delay (t(35) = 4.454, P < 0.001), and this finding survived the Bonferroni correction (α = 0.017; 0.05/3 comparisons). LDI scores did not differ significantly between the (i) no delay and awake quiescence delay groups (t(36) = −1.602, P = 0.118), or (ii) no delay and perceptual task delay groups (t(35) = 1.876, P = 0.069) - see Fig. 2D.

If the benefit of awake quiescence delay over perceptual task delay in the LDI measure reflects a difference in participants’ ability to correctly discriminate lures from targets, an interaction should be observed between delay condition group (quiescence vs. task) and response type to lure items (“old” vs. “similar”). Specifically, a greater proportion of correct “similar” responses to lures (i.e. correct identification of lures) would be expected in the quiescence group and a greater proportion of incorrect “old” responses to lures (i.e. incorrectly mistaking lures for targets) would be expected in the task group. In line with this prediction a 2 × 2 ANOVA revealed a significant interaction between delay condition group and response type to lure items (F(1,35) = 9.030, P = 0.005, ηρ² = 0.205). Pairwise comparisons of the proportion of “old” and “similar” responses to lure items – see Fig. 2B – confirmed that participants in the perceptual task delay provided a significantly higher proportion of (incorrect) “old” responses to lure items than those in the awake quiescence delay (t(35) = −2.395, P = 0.022). In contrast, participants in the awake quiescence delay provided a significantly higher proportion of (correct) “similar” responses to lure items than those in the perceptual task delay (t(35) = 3.584, P = 0.001). Moreover, participants in the awake quiescence delay provided a significantly smaller proportion of (incorrect) “new” responses to lure items (t(35) = −3.141, P = 0.003) and (incorrect) “similar” responses to foil items (t(35) = −2.613, P = 0.013) than the perceptual task delay. No other comparisons reached significance (all P > 0.05). When using a Bonferroni-corrected alpha level of 0.006 (0.05/9 comparisons), only the differences in “similar” responses to lures and “new” responses to lure items remained significant.

Performance on standard recognition, which was calculated as the proportion of “old” responses to target items minus the proportion of “old” responses to foils, was near ceiling across groups (see Fig. 2E). This indicates that all participants encoded and retained (at least) gist memory of most presented target items. Although there was no significant main effect of delay group in this measure (F(2,53) = 2.600, P = 0.084, ηρ² = 0.089), those who experienced no delay between encoding and testing were significantly better at discriminating targets from foil items than those who experienced 10 mins of a perceptual task (t(35) = 2.145, P = 0.039), though this finding did not survive the Bonferroni correction (α = 0.017; 0.05/3 comparisons). There were no significant differences in standard recognition performance between (i) the no delay and awake quiescence delay groups (t(36) = 1.424, P = 0.163), or (ii) the awake quiescence delay and perceptual task delay groups (t(35) = 1.033, P = 0.309).

As reported above, the LDI and standard recognition scores of the MST rely on the subtraction of probabilities. While this method has been shown to be sensitive to various experimental manipulations^28–30,34^, concerns have been raised regarding the method by which these measures are calculated^33^. These concerns relate to the use of proportional values in measurement calculation due to inconsistencies across studies and an inability to directly compare against signal detection theory analyses^33^. Thus, we examined whether our results held when using signal detection theory by computing d’ scores using the standard formula: z(hit rate)-z(false alarm rate). Specifically, we computed traditional memory sensitivity d’ scores for the standard recognition measure [z(proportion of old responses to targets)-z(proportion of old responses to foils)] in order to show how well old items were discriminated from new items. We also computed target vs. lure discrimination scores [z(proportion of old responses to targets)-z(proportion of old responses to lures)] to show how well old targets were discriminated from similar lures. See Table 1 for group means of these two measures. Critically, analysis of these new signal detection theory scores replicated the findings of our proportional scores. All significant results in proportional analyses were significant in our signal detection theory analyses (see below).

**Table 1:** Signal detection theory scores for the no delay, 10 min quiescence and 10 min task delay condition groups.

	Target vs. foil discrimination score (d’)	Target vs. lure discrimination score
No delay	3.53 (0.50)	1.63 (0.56)
10 min quiescence	3.23 (0.62)	1.67 (0.35)
10 min task	3.07 (0.72)	1.27 (0.62)

Standard deviations are shown in parentheses.

For the traditional memory sensitivity d’ scores, we found no significant main effect of delay condition group (F(2,53) = 2.656, P = 0.080, ηρ² = 0.091). Pairwise comparisons revealed no significant difference in d’ scores between the quiescence and task groups (t(35) = 0.743, P = 0.463), or no delay and quiescence groups (t(36) = −1.627, P = 0.112). We did find a significant difference between the no delay and task groups (t(35) = −2.265, P = 0.030), though this finding did not survive the Bonferroni correction (α = 0.017; 0.05/3 comparisons).

For the target vs. lure discrimination scores, we found a significant main effect of delay group (F(2,53) = 3.249, P = 0.047, ηρ² = 0.109). Pairwise comparisons confirmed a significant difference between the quiescence and task delay groups (t(35) = 2.405, P = 0.022). There was no significant difference between the no delay and quiescence delay groups (t(36) = −0.249, P = 0.804), or no delay and task delay groups (t(35) = 1.850, P = 0.072). These findings are in keeping with our results when participants’ ability to discriminate lures from targets was computed using proportional scores (Lure Discrimination Index, LDI; see above), though it is noted that, unlike when using the LDI measure, the significant difference between the quiescence and task delay groups did not survive Bonferroni correction (α = 0.017; 0.05/3 comparisons).

**Figure 3:**
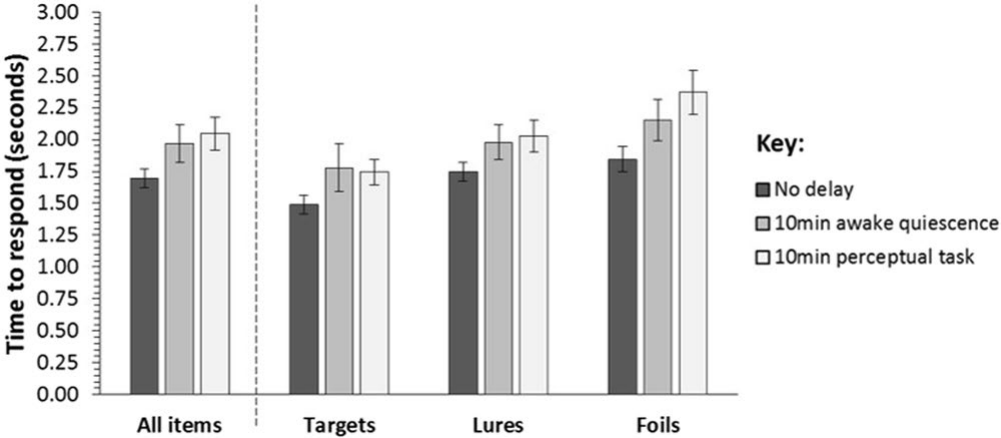
Mean response times in the Mnemonic Similarity Test for the no delay (N = 19), 10 min awake quiescence delay (N = 19), and 10 min perceptual task delay (N = 18) groups, across all target, lure, and foil items (*left*). Mean response times for each delay group broken down by responses to target, lure, and foil items (*right*). In all cases, error bars show the standard error of the mean.

The groups’ response times during the testing phase did not differ significantly (F(2,53) = 2.415, P = 0.099, ηρ² = 0.084) - see Fig. 3. However, we found that, overall, participants’ responses differed significantly for item type (target vs. lure vs. foil) (F(2,106) = 33.914, P < 0.001, ηρ² = 0.390). As shown in Fig. 3, there was an overall increase in the time it took participants to respond between target and lure items (t(55) = −4.672, P < 0.001) and lure and foil items (t(55) = −3.940, P < 0.001). As a result, there was also a significant difference in response times between target and foil items (t(55) = −7.304, P < 0.001), where participants were slower to respond to the latter. All findings survived Bonferroni correction (α = 0.017; 0.05/3 comparisons). There was no significant interaction between item type and delay group (F(4,106) = 1.463, P = 0.219, ηρ² = 0.052).

## Discussion

Our results show that participants who rested quietly for 10 minutes (awake quiescence) after viewing photos of everyday items were subsequently better at discriminating these target items from visually similar lure items, relative to those who were engaged in an unrelated perceptual task for 10 minutes. This finding suggests that awake quiescence protects the fine detail of new memories, bestowing on them a higher resolution than they would otherwise have after a filled delay.

The effect of the awake quiescence delay, relative to the perceptual task delay, cannot be explained away by potential between-group differences in participants’ background or encoding performance because the groups were well matched on these measures (see supplementary information for analyses). Furthermore, it is unlikely that this effect can be accounted for merely by intentional rehearsal of items during the 10-minute quiescence delay because the effect remained even when we excluded 4 participants who reported thinking about the items while resting (see supplementary information for analyses). In fact, the key group difference emerged in the LDI measure (see below), and it is unlikely that thinking about a presented item, e.g. a tree, during the rest period would have allowed participants to discriminate a similar lure tree from a previously encoded target tree (see Fig. 1). In addition, we observed no between-group differences in the time that it took participants to respond to items presented during testing. Therefore, it is also unlikely that the difference in memory performance between the awake quiescence and perceptual task delays can be accounted for by differences in the groups’ ability to retrieve stored representations. Moreover, in light of concerns regarding the use of proportions in recognition memory measures^33^, results were replicated when analysing the scores computed via signal detection theory (see results for details).

We hypothesise that superior LDI scores in those who experienced awake quiescence, relative to the perceptual task, can be explained by better/increased *consolidation* during rest. In particular, our findings suggest that rest-related consolidation protects the fine detail of new memories. This, we propose, resulted in participants in the awake quiescence delay having a better subsequent ability to discriminate similar lures from encoded targets, relative to those in the task delay. Our consolidation hypothesis resonates with other recent findings that suggest that successful discrimination of target items and lures is dependent, not only on the successful encoding of memory representations, but also on the early-stage consolidation of these newly encoded representations^30,31^. Specifically, Borota *et al*. (2014) showed higher 24-hour MST LDI scores in participants who consumed caffeine immediately after encoding than in participants who consumed a placebo^30^. The superior LDI score could not be explained by a retrieval account because no superior LDI score was demonstrated in control participants who consumed the caffeine immediately prior to the 24-hour test only. Instead, Borota *et al*.^30^ hypothesise that caffeine enhances the consolidation of new memories, thus facilitating the discrimination of lures from target items. The mechanisms by which caffeine does so remain unknown^30^. Moreover, Doxey *et al*. (2018) recently reported higher MST LDI scores in participants who slept for an extended period after encoding than in participants who spent the equivalent time in active wakefulness (e.g. going about daily activities)^35^. They hypothesise that the benefit of sleep in the MST reflects superior consolidation during sleep than during active wakefulness^35^. In our study, it is possible that awake quiescence protected the fine detail of new memories by providing a state of reduced sensory input and task engagement. These factors are hypothesised to impact automatic consolidation-related neural reactivation, and their reduction might therefore be conducive to memory consolidation^16–18^.

In contrast to the LDI measure, performance in the standard recognition measure, which probed participants’ ability to endorse targets and reject foils, was not significantly different between delay groups (see Fig. 2E). We did find a significantly lower standard recognition score in the perceptual task delay than the no delay group, but this apparent forgetting did not survive corrections for multiple comparisons. Similar findings of a significant effect in the MST LDI, but not in standard recognition, have been reported in a range of studies applying the MST, including the aforementioned studies on the effects of post-encoding caffeine^30^ and sleep^35^, studies examining the effect of healthy and pathological ageing^28,34,36^, hippocampal damage^37^, as well as acute exercise^38^. Together, these findings demonstrate that standard (old/new) recognition performance can be achieved via gist memory alone, in the absence of detailed target memories^32,33^, and that the various factors above do not affect gist memory substantially.

In our study, performance in the standard recognition measure was near ceiling across groups. Therefore, it cannot be inferred whether the conditions experienced during the delay period affected the consolidation of gist memory for target items. Nevertheless, this near-ceiling performance in the standard recognition measure provides greater insight into the scores observed in the LDI measure. It suggests that, irrespective of delay condition, participants retained gist memory for most presented targets. This was not the case for detail memory of the targets. Participants retained less detail memory (see Fig. 2B and D), and critically, our results indicate that the behavioural conditions experienced during the 10-minute delay significantly affected the consolidation of this detail memory of targets.

It is of note that whereas the awake quiescence delay group’s LDI score was significantly higher than that of the perceptual task delay, neither groups’ LDI scores differed significantly from that of the No delay group. However, Fig. 2D suggests tentatively that memory detail increased slightly during the awake quiescence delay and decreased more substantially during the perceptual task delay, albeit not significantly. It is possible that these effects would have been more robust and reached significance had we (i) imposed a longer delay between the encoding and test phase, and/or (ii) applied a within-subjects design, which would have provided more statistical power.

In summary, we provide the first evidence that rest-related consolidation protects the fine detail of new memories. This provides us with higher resolution memories and supports our ability to discriminate recently encoded memories from similar representations. Our findings also support the view that consolidation benefits from states of reduced sensory processing more generally, rather than being restricted to sleep^1,16^. Future work should examine the neurocognitive mechanisms of rest-related memory consolidation, with a focus on unveiling the factors implicated in the consolidation of detail memory.

## Methods

### Subjects

Sixty young adults (35 females, 25 males; mean age = 21.77 years, SD = 2.90, age range: 18–31 years) were recruited as participants. All participants had normal or corrected-to-normal visual acuity and no known premorbid psychiatric or neurological disorders that might affect performance. This research was approved by Heriot-Watt University’s School of Life Sciences Research Ethics Committee (Ref: 2015-140) and all procedures adhered to the appropriate ethical principles for research in humans. All participants were briefed and provided their informed consent in writing prior to their participation. Twenty participants were pre-experimentally allocated pseudo-randomly to each of our three delay condition groups. Three participants (1 × awake quiescence, 2 × perceptual task) scored +2 SDs from their respective group means in our LDI measure, while one participant scored +2 SDs from their group mean in the standard recognition measure (1 × no delay), and were thus deemed as outliers and removed from our analyses. Thus, group Ns in the analyses reported below were as follows: no delay = 19; awake quiescence delay = 19; perceptual task delay = 18. It is however worth noting that no significant results changed when all recruited participants (N = 60) were included in our analyses.

### Design

We employed a between-subjects design to examine retention of photographs of everyday items. The experiment took place in a single session, divided into an encoding phase, a delay phase, and a testing phase. Our experimental manipulation occurred during the delay phase (see Fig. 1).

### Materials

Our paradigm used a modified (shortened) version of the Mnemonic Similarity Task^28^, which was combined with our sensitive memory paradigm that has been shown to detect positive rest-related effects in memory consolidation^24,26,27^. The computerised task used to administer our procedure was developed and run using PsychoPy (version 1.83.01) via a Python-coded script^39^. The task was presented via a 22-inch wide screen computer monitor. Visual instructions were presented on the computer screen during the procedure. Stimuli (photos of everyday items) in the encoding and testing phases were presented in the centre of the screen on all occasions. Responses during encoding and testing phases were collected from participants via keyboard input.

### Procedure

#### Encoding phase

Participants were pre-experimentally informed that they were taking part in a study investigating how humans make judgements about everyday items. They were not informed that they would experience one of three delay conditions or a subsequent memory test for the presented items. This incidental encoding procedure has been used previously^28^, and was particularly relevant in our study because it minimises the likelihood of mnemonic strategies, e.g. active rehearsal of items during the delay period, in particular the awake quiescence delay^18,24,26^.

Participants were presented a total of 60 photos of a range of unique everyday items (see Fig. 1 for examples). Each item was presented as a standalone item on a white background. As in previous work^28^, each item was presented for a duration of 2 seconds, with an inter-stimuli crosshair (+) appearing in the centre of the screen for 0.5 seconds (total duration of encoding phase = 150 seconds). Thus, all participants received identical treatment and exposure to stimuli during encoding. When presented an item, participants were required to make a judgment as to whether they believed the item would typically be found indoors or outdoors. For example, if presented a photo of a sofa, this item would typically be found indoors, but if presented a photo of a tree, this item would typically be found outdoors. Participants input responses via the ‘z’ (indoors) and ‘m’ (outdoors) keys on the keyboard. Participants were instructed that some items may be ambiguous in their typical location (i.e. may be found indoors and outdoors), but that they should respond as quickly as possible and respond with their first instinct.

#### Delay phase

During the delay phase, participants experienced one of three delay conditions, where they either (i) moved immediately from the encoding phase to the testing phase (i.e. no delay) (N = 20), (ii) rested wakefully for 10 minutes between the encoding and testing phases (i.e. awake quiescence delay) (N = 20), or (iii) performed an unrelated perceptual task (a spot-the-difference game) for 10 minutes between the encoding and testing phases (i.e. perceptual task delay) (N = 20). Participants assigned to the *awake quiescence* condition were asked to sit quietly in the dimly-lit testing room and relax while the experimenter left the room to “set up the next section of the experiment”^18^. Care was taken to ensure that the testing room was devoid of any rich visual and/or audible sensory cues to minimise sensory information, and thus the disruption of consolidation. Participants assigned to the *perceptual task* delay were asked to play a visual spot the difference game^18^. Participants performed a total of 30 spot-the-difference trials in silence, each 20 second in duration. A trial consisted of the presentation of a pair of real-world photos on the computer screen (see Fig. 1 for examples). Photos were identical other than for two discrete differences. Participants were instructed to search for these two differences, and to silently point toward them if discovered. The experimenter sat behind the participant during this task and scored the number of differences that the participant correctly identified. There was no overlap between the contents of the spot-the-difference photos and the MST photos of everyday items. The no-delay condition was included as a baseline measure of recognition performance (i.e. immediate memory test).

#### Testing phase

Following the delay phase, all participants performed a modified (fewer items) version of the Mnemonic Similarity Task (MST)^28^, which probed the fine detail of memories for stimuli presented during the encoding phase. In this test, participants were sequentially visually presented 90 photos of everyday items in a random order which was the same across participants. Of these photos, 30 were *old*, i.e. were identical to those presented during encoding (targets), 30 were *similar* (visually similar items from the same semantic category) to the remaining photos presented during encoding (lures), and 30 were *new* to those presented during encoding (foils) - see Fig. 1 for examples. Participants were informed that, like in the first part of the study (Encoding phase), they would be visually presented a set of photos of everyday items in the centre of the computer screen. However, on this occasion, rather than performing an “indoor/outdoor” judgment-making task, their memory for the earlier presented items would be probed. They were instructed that presented items would be either (i) visually identical to those presented earlier (old targets), (ii) visually similar to those presented earlier (similar lures), or (ii) brand new and not presented earlier (new foils). Using the computer keyboard, they were asked to provide an ‘old’ (visually identical target item; ‘z’ key), ‘similar’ (visually similar lure item; ‘v’ key), or ‘new’ (new foil item; ‘m’ key) response. Participants were informed that there was no time limit to provide a response, and they should respond as accurately as possible. Onscreen instructions regarding which keys corresponded to which response (e.g. ‘z’ = old) were shown at all times during encoding and testing. To reduce the possibility of order presentation effects, target items were always the oddly numbered items presented during the encoding phase (i.e. 1, 3, 5, 7, 9…etc) and lure items were related to the evenly number items presented during the encoding phase (i.e. 2, 4, 6, 8, 10…etc). The same order of items was presented to all participants during encoding. These 30 targets and 30 lure items were then combined with 30 foil items and then ordered randomly using the random number generation tool in Microsoft Excel. The same random order of items was presented to all participants during testing.

### Scoring

Performance in the MST was scored as in previous work^28,30,34,40^. Firstly, the total number of *old*, *similar*, and *new* responses for the 30 targets, 30 lures, and 30 target items was extracted and converted to proportion scores (each score/30). From these proportion scores, two key memory measures were calculated: (i) a standard recognition score, and a Lure Discrimination Index (LDI) score^28^. The standard recognition score is calculated as the proportion of “old” responses to targets minus the proportion of “old” responses to foils, and thus reflects a person’s ability to correctly endorse targets, while rejecting foils. The LDI score is calculated as the proportion of “similar” responses to lures minus the proportion of “similar” responses to foils, and thus reflects a person’s ability to discriminate lures from targets, while controlling for any response biases^28,36^. In addition to these proportional scores, we examined performance in line with signal detection theory by computing d’ scores using the standard formula: z(hit rate)-z(false alarm rate). Specifically, we computed traditional memory sensitivity d’ scores for the standard recognition measure [z(proportion of old responses to targets)-z(proportion of old responses to foils)] in order to show how well old items were discriminated from new items. We also computed target vs. lure discrimination scores [z(proportion of old responses to targets)-z(proportion of old responses to lures)] to show how well old targets were discriminated from similar lures. Finally, we extracted the time that it took participants to respond during encoding and testing to check for any potential group differences in encoding and/or retrieval.

### Statistical analyses

Analyses were performed using SPSS Statistics 19 (copyright IBM Corp., NY, USA), with the alpha level set to 0.05. ANOVAs with between-subject factor delay condition (no delay vs. awake quiescence delay vs. perceptual task delay) were performed to examine group differences in encoding, LDI and standard recognition scores and test response times. Two-tailed independent t-tests were conducted to examine group differences between pairs of groups. Paired t-tests were conducted to examine within-subject differences in responses to different item types. Bonferroni corrections for multiple comparisons were applied post-hoc to avoid Type 1 error accumulation.

### Data availability

The data that support the findings of this study are available from the corresponding author upon request.

## Electronic supplementary material

Supplementary Information

## References

[1] Wixted JT (2004). The psychology and neuroscience of forgetting. Annu. Rev. Psychol..

[2] Dudai Y (2004). The neurobiology of consolidations, or, how stable is the engram?. Annu. Rev. Psychol..

[3] Deuker L (2013). Memory consolidation by replay of stimulus-specific neural activity. J. Neurosci..

[4] Carr MF, Jadhav SP, Frank LM (2011). Hippocampal replay in the awake state: a potential substrate for memory consolidation and retrieval. Nat. Neurosci..

[5] Oudiette D, Antony JW, Creery JD, Paller KA (2013). The role of memory reactivation during wakefulness and sleep in determining which memories endure. J. Neurosci..

[6] Karlsson MP, Frank LM (2009). Awake replay of remote experiences in the hippocampus. Nat. Neurosci..

[7] Ramadan W, Eschenko O, Sara SJ (2009). Hippocampal sharp wave/ripples during sleep for consolidation of associative memory. PLoS One.

[8] Gupta AS, van der Meer MAA, Touretzky DS, Redish AD (2010). Hippocampal replay is not a simple function of experience. Neuron.

[9] Staresina BP, Alink A, Kriegeskorte N, Henson RN (2013). Awake reactivation predicts memory in humans. Proc. Natl. Acad. Sci..

[10] Foster DJ, Wilson MA (2006). Reverse replay of behavioural sequences in hippocampal place cells during the awake state. Nature.

[11] Ego-Stengel V, Wilson MA (2011). Disruption of ripple-associated hippocampal activity during rest impairs spatial learning in the rat. Hippocampus.

[12] Girardeau M, Benchenane K, Wiener S, Buzsáki G, Zugaro M (2009). Selective suppression of hippocampal ripples impairs spatial memory. Nat. Neurosci..

[13] Jadhav SP, Kemere CP, German W, Frank LM (2012). Awake Hippocampal Sharp-Wave Ripples Support Spatial. Memory..

[14] Maingret N, Girardeau G, Todorova R, Goutierre M, Zugaro M (2016). Hippocampo-cortical coupling mediates memory consolidation during sleep. Nat. Neurosci..

[15] Schönauer, M. *et al*. Decoding material-specific memory reprocessing during sleep in humans. *Nat*. *Commun*. **8**, (2017). 10.1038/ncomms15404 PMC5442370 28513589

[16] Mednick SC, Cai DJ, Shuman T, Anagnostaras S, Wixted JT (2011). An opportunistic theory of cellular and systems consolidation. Trends Neurosci..

[17] Alber J, Della Sala S, Dewar M (2014). Minimising interference with early consolidation boosts 7-day retention in amnesic patients. Neuropsychology.

[18] Dewar M, Alber J, Butler C, Cowan N, Della Sala S (2012). Brief wakeful resting boosts new memories over the long term. Psychol. Sci..

[19] Gaskell MG (2014). Sleep Underpins the Plasticity of Language Production. Psychol. Sci..

[20] Wamsley EJ, Tucker MA, Payne JD, Stickgold R (2010). A brief nap is beneficial for human route-learning: The role of navigation experience and EEG spectral power. Learn. Mem..

[21] Tamminen J, Payne JD, Stickgold R, Wamsley EJ, Gaskell MG (2010). Sleep spindle activity is associated with the integration of new memories and existing knowledge. J. Neurosci..

[22] Walker MP, Stickgold R (2010). Overnight alchemy: sleep-dependent memory evolution. Nat. Rev. Neurosci..

[23] Craig M, Sala SD, Dewar M, Della Sala S, Dewar M (2014). Autobiographical thinking interferes with episodic memory consolidation. PLoS One.

[24] Dewar M, Cowan N, Della Sala S (2007). Forgetting due to retroactive interference: a fusion of Müller and Pilzecker’s (1900) early insights into everyday forgetting and recent research on anterograde amnesia. Cortex.

[25] Craig M, Dewar M, Della Sala S, Wolbers T (2015). Rest Boosts the Long-term Retention of Spatial Associative and Temporal Order Information. Hippocampus.

[26] Craig M, Dewar M, Harris MA, Della Sala S, Wolbers T (2016). Wakeful rest promotes the integration of spatial memories into accurate cognitive maps. Hippocampus.

[27] Dewar M, Alber J, Cowan N, Della Sala S (2014). Boosting Long-Term Memory via Wakeful Rest: Intentional Rehearsal Is Not Necessary, Consolidation Is Sufficient. PLoS One.

[28] Stark SM, Yassa MA, Lacy JW, Stark CEL (2013). A task to assess behavioral pattern separation (BPS) in humans: Data from healthy aging and mild cognitive impairment. Neuropsychologia.

[29] Yassa MA (2010). High-resolution structural and functional MRI of hippocampal CA3 and dentate gyrus in patients with amnestic Mild Cognitive Impairment. Neuroimage.

[30] Borota D (2014). Post-study caffeine administration enhances memory consolidation in humans. Nat. Neurosci..

[31] Bekinschtein P (2013). BDNF in the dentate gyrus is required for consolidation of ‘pattern-separated’ memories. Cell Rep..

[32] Migo E, Montaldi D, Norman KA, Quamme J, Mayes A (2009). The contribution of familiarity to recognition memory is a function of test format when using similar foils. Q. J. Exp. Psychol. (Hove)..

[33] Loiotile RE, Courtney SM (2015). A signal detection theory analysis of behavioral pattern separation paradigms. Learn. Mem..

[34] Stark SM, Stark CEL (2017). Age-related deficits in the mnemonic similarity task for objects and scenes. Behav. Brain Res..

[35] Doxey, C. R., Hodges, C., Bodily, T., Muncy, N. M. & Kirwan, C. B. The Effects of Sleep on the Neural Correlates of Pattern Separation. *Hippocampus* 1–41 (2017). 10.1002/hipo.22814 29149767

[36] Stark SM, Stevenson R, Wu C, Rutledge S, Stark CEL (2015). Stability of age-related deficits in the mnemonic similarity task across task variations. Behav. Neurosci..

[37] Kirwan CB (2012). Pattern separation deficits following damage to the hippocampus. Neuropsychologia.

[38] Suwabe, K. *et al*. Acute Moderate Exercise Improves Mnemonic Discrimination in Young Adults. **234**, 229–234 (2017). 10.1002/hipo.22695 PMC5927776 27997992

[39] Peirce JW (2008). Generating Stimuli for Neuroscience Using PsychoPy. Front. Neuroinform..

[40] Bakker A (2012). Reduction of Hippocampal Hyperactivity Improves Cognition in Amnestic Mild Cognitive Impairment. Neuron.

