# Supplementary material for: Rest-related consolidation protects the fine detail of new memories
Source: Sci Rep. 2018 May 1;8:6857. doi: 10.1038/s41598-018-25313-y (PMC5931514; doi:10.1038/s41598-018-25313-y)
Supplement: Supplementary file 1 — Supplementary Information [file 41598_2018_25313_MOESM1_ESM.docx]

***Supplementary information***

**Rest-related consolidation protects the fine detail of new memories**

**Michael Craig* & Michaela Dewar**

**Department of Psychology, School of Social Sciences, Heriot-Watt University, Edinburgh, EH14 4AS, United Kingdom**

**^*^Correspondence regarding this article should be sent to Dr Michael Craig at m.craig@hw.ac.uk.**

**Nature Scientific Reports - Supplementary Results**

# Background measures

# Results demonstrated that participants in our three delay groups did not differ significantly in (i) gender ratio (no delay: 7M:12F; awake quiescence delay: 10M:9F; perceptual task delay: 8M:10F; Chi-squared test: *x^2^*(55) = 0.959, P = .619), (ii) age (F(2,53) = 0.713, P = .495, ηρ² = .026), and (iii) total number of years spent in education (F(2,53) = 0.213, P = .809, ηρ² = .008). Planned pairwise comparisons confirmed no significant between-group differences in the above background measures (all P > .255).

# Encoding phase

# Participants performed an indoor vs. outdoor judgment task during the encoding phase, and thus, the to-be-retained stimuli (photos of everyday items), were incidentally encoded. While we were not specifically interested in participants’ judgments in this task, we recorded the time that it took participants to respond, in order to ensure that groups were well-matched in their performance during encoding. On average, participants responded to in excess of 95% of trials (57/60 trials), where the proportion of responses to trials did not differ between groups (no delay: 95.44%, SD = 0.04%; awake quiescence delay: 95.18%, SD = 0.06%; perceptual task delay: 96.11%, SD = 0.04%; F(2,53) = 0.182, P = .834, ηρ² = .007). The mean time that participants took to respond to trials also did not differ between groups (no delay: 0.95 seconds, SD = 0.31; awake quiescence delay: 0.99 seconds, SD = 0.33; perceptual task delay: 1.00 seconds, SD = 0.24; F(2,53) = 0.157, P = .855, ηρ² = .006). Thus, groups were well matched in their pre-delay condition performance, though we acknowledge that this does not completely rule out potential encoding-related differences between groups.

# Post-experimental reports

# A small number of participants (N=4) in the awake quiescence delay group reported either spontaneous (N=2) or intentional (N=2) thoughts pertaining to the initially encoded items during the 10 minute rest delay. However, as in previous work^2–4^, no results changed when these participants were excluded from analyses. Furthermore, we made a conscious effort to ensure that there was little semantic/visual overlap between the presented items and the stimuli used in the perceptual task delay, and indeed, only two participants in this condition reported that the spot-the-difference images triggered memories for the to-be-retained stimuli. Importantly, as above, when we removed these participants from our analyses, no results changed. Therefore, it is unlikely that the LDI difference between the awake quiescence and perceptual task delays was attenuated by reminders of the objects during the spot-the-difference task.
